# Human and Murine Evidence for Mechanisms Driving Autoimmune Photosensitivity

**DOI:** 10.3389/fimmu.2018.02430

**Published:** 2018-10-23

**Authors:** Sonya J. Wolf, Shannon N. Estadt, Johann E. Gudjonsson, J. Michelle Kahlenberg

**Affiliations:** ^1^Division of Rheumatology, Department of Internal Medicine, University of Michigan, Ann Arbor, MI, United States; ^2^Immunology Program, University of Michigan, Ann Arbor, MI, United States; ^3^Department of Dermatology, University of Michigan, Ann Arbor, MI, United States

**Keywords:** ultraviolet light, lupus (SLE), photosensitivity, cytokines, apoptosis

## Abstract

Ultraviolet (UV) light is an important environmental trigger for systemic lupus erythematosus (SLE) patients, yet the mechanisms by which UV light impacts disease are not fully known. This review covers evidence in both human and murine systems for the impacts of UV light on DNA damage, apoptosis, autoantigen exposure, cytokine production, inflammatory cell recruitment, and systemic flare induction. In addition, the role of the circadian clock is discussed. Evidence is compared in healthy individuals and SLE patients as well as in wild-type and lupus-prone mice. Further research is needed into the effects of UV light on cutaneous and systemic immune responses to understand how to prevent UV-light mediated lupus flares.

## Introduction

Ultraviolet (UV) light is a pervasive environmental exposure with pleotropic effects on the skin. Sensitivity to UV light is a shared feature of several autoimmune diseases, including systemic and cutaneous lupus erythematosus (SLE and CLE, respectively), dermatomyositis, and occasionally Sjögren's syndrome. The reported frequency of photosensitivity reaches up to 93% in lupus patients, depending on the underlying disease pathology (1–3), is suggested to be around 50% in dermatomyositis (4), and is poorly documented but reported anecdotally in Sjögren's syndrome. Patients with these disorders can manifest with varied skin reactions including erythema, inflammatory lesions to moderate exposures, and severe skin inflammation and systemic disease flares (especially in SLE) to larger exposures (5, 6). Despite the clinical acknowledgement of photosensitivity, the mechanistic reasons for sensitivity to UV exposure remain unclear. In this review, we discuss the effects of UV light on the skin in both human and murine systems and how disease-associated changes may promote abnormal reactivity and increased inflammation to UV exposure.

### Human healthy and autoimmune skin responses to UV

UV light falls in the spectrum between visible light and gamma irradiation. UVA, UVB, and UVC are divided based on wavelength (UVA = 400–320 nm; UVB = 320–280 nm; UVC = 280–100 nm), with shorter wavelengths associated with higher energy effects. In general the longer wavelengths, such as UVA, which has been shown to have therapeutic potential in SLE patients (7–9), penetrate more deeply in the skin, reaching the dermis, whereas UVB is absorbed almost entirely by the keratinocytes of the epidermis (10). UVC rarely reaches the skin as it is primarily absorbed by atmospheric ozone. Following UV exposure, the keratinocytes act as first responders, trigging inflammatory cytokine, and chemokine production. If the UV exposure is substantial enough, keratinocytes also undergo apoptosis.

### UV-induced DNA damage and apoptosis

UV exposure induces DNA damage, which can result in the formation of dimeric photoproducts involving neighboring pyrimidine bases (11–13). UV irradiation can also cause an accumulation of reactive oxygen species (ROS) in keratinocytes that results in oxidative damage to DNA, lipids, and proteins and can ultimately induce apoptosis (11, 13–15). Of importance, the oxidative damage to DNA bases can lead to formation of 8-hydroxyguanosine (8-OHG) which has been shown to be immunogenic in patients with lupus erythematosus (LE) and abundantly present in UV-induced LE lesions (16–18).

DNA methylation is also altered by UV exposure, as several groups have shown that UVB irradiation decreases DNA methylation in CD4+ T cells of patients with SLE by inhibiting the catalytic activity of DNA methyltransferase 1 (DNMT1) (19–21). A positive correlation between the levels of DNMT1 expression and global DNA methylation is seen in SLE patients suggesting that aberrant expression of this enzyme is involved in these methylation changes (22). This suppression seems to be mediated by the UVB-induced activation of the aryl hydrocarbon receptor (AhR) which subsequently inhibits silent mating type information regulation 2 homolog 1 (SIRT1) and DNMT1 activity (21). SIRT1 and AhR have both been suggested to be involved in the pathogenesis of lupus and may provide a link between disease and photosensitivity (21, 23, 24). UV light can cause formation of tryptophan photoproducts that are able to act as AhR agonists (25). Furthermore, activation of AhR correlates with cutaneous expression of interleukin-22 (IL-22), a cytokine that, in the presence of IFNα, can activate STAT1 and upregulate CXCL9 and CXCL10 further contributing to inflammation (24, 26).

High mobility group box 1 protein (HMGB1) is regulated by UV exposure, and serves as a DNA binding protein that plays a role in regulation of transcription. During instances of injury or inflammation, activated macrophages, natural killer cells, and dendritic cells can secrete HMGB1 to help coordinate immune responses by recruiting leukocytes, augmenting production of pro-inflammatory cytokines, and activating NFkB through RAGE and Toll-like receptors (TLRs) (27). HMGB1 can also be released by cells undergoing necrosis or apoptosis and subsequently enhance inflammatory responses (27, 28). SLE patients have increased levels of HMGB1 that correlate with both levels of pro-inflammatory cytokines, including TNFα, IL-6, and IL-1β, and disease activity (29, 30). Following UVB exposure, HMGB1 is released from SLE keratinocytes at an increased rate and in an apoptosis-related manner, which may thus contribute to the development of UV-induced inflammation and lead to skin lesion formation (31).

Following DNA damage, apoptosis is induced in keratinocytes. Research has explored whether SLE patients are more susceptible to this DNA damage. One study, which used immunohistochemistry for cleaved caspase-3, found no difference in epidermal apoptotic cells 24 h after 1x and 2x minimal erythema UV dose in SLE patients (32). However, others have identified increases in apoptotic bodies in the skin of CLE patients after UV treatment when compared with control skin (33). Another group identified increases in TUNEL staining in SLE vs. control skin after UVB; this was also true when SLE vs. normal keratinocytes were treated with UVB *in vitro* (34). It is important to consider, however, that TUNEL staining may represent other forms of cell death in addition to apoptosis (35–38). CLE lesions themselves also demonstrate increased TUNEL staining, which supports a cell-death phenotype in lesions *in vivo* (39).

### UV- induced autoantigen exposure

UVB irradiation can result in the translocation of Ro/SSA and La/SSB antigens from the nucleus and cytoplasm to the surface of apoptotic human keratinocytes rendering them susceptible to binding by their respective circulating autoantibodies (14, 40–42). Photosensitivity of patients correlates with both the presence of anti-Ro and anti-La autoantibodies and the increased expression of Ro/SSA and La/SSB in keratinocytes (43–46). Both photoprovoked and spontaneous CLE lesions as well as UV-irradiated patient-derived primary keratinocytes show increased Ro52 expression (47).

Another autoantigen suggested to be involved in lupus is interferon-inducible protein 16 (IFI16), a DNA binding protein with diverse roles that is normally localized to the nucleus (48). SLE patient serum often has high titers of anti-IFI16 antibodies, with one study finding these antibodies could be detected in 29% of sera collected from 374 SLE patients (49, 50). Upon UVB irradiation of keratinocytes, IFI16 is redistributed to the cytoplasm and the extracellular space, leaving it exposed for possible immune recognition by autoantibodies and potentially contributing to the inflammatory environment associated with photosensitivity (50). UVB has also been shown to increase autoantibody binding to other autoantigens including Sm, RNP, Ku, and ribosomal-P (51–53). In particular, anti-Sm and anti-ribosomal-P antibodies are strongly associated with photosensitivity and disease activity in lupus patients (53–55).

Following induction of apoptosis, reduced clearance of apoptotic cells in lupus skin has also been suggested to contribute to induction of inflammatory lesions, likely through increased exposure to auto-antigens. Most studies support dysfunctional and reduced phagocytosis in SLE patients, ultimately resulting in reduced clearance of apoptotic cells (56–58). Part of this phenotype may be regulated by UVB as HMGB1can skew macrophage polarization toward an M1-like phenotype diminishing their ability to phagocytose apoptotic cells (59).

Another mechanism regulating apoptotic clearance is opsonization by complement. Homozygous deficiency of complement proteins of the classical pathway is associated with SLE pathogenesis (60, 61). The strongest associations are seen with proteins involved in the earliest steps of the pathway including C1q and C4, with as many as 75–90% of patients with a homozygous deficiency of these proteins reported to have SLE or lupus-like disease (61). A study of Swedish SLE patients found that 16% had homozygous C4A deficiency and photosensitivity was more common among these patients (62). Mechanisms of lupus risk associated with complement deficiencies may be related to decreased apoptotic clearance. For example, C1q binds to the nucleolus of cells undergoing UV-induced apoptosis resulting in activation of C1r/C1s proteases that under normal circumstances facilitate degradation of these potential autoantigens (63–66). In addition, C1q provides anti-inflammatory functions in macrophages (67) and suppresses type I interferon (IFN) production (68, 69), an important component of CLE lesions. Importantly, single nucleotide polymorphisms in C2, particularly in Chinese SLE patients, are strongly associated with photosensitivity among other clinical manifestations of disease (70). These data indicate that defective complement pathways resulting in deficient clearance of apoptotic cells are likely involved in increased photosensitivity and lesion development in lupus patients.

### UV-induced inflammation

#### Cytokines

UV exposure may have repressive or activating functions on cytokine production depending on the context. In normal keratinocytes, UVB upregulates suppressor of cytokine signaling (SOCS) 1 and 3 and downregulates activation of STAT1, resulting in resistance to activation effects of IFN-γ (71, 72). Following UVB exposure, cutaneous production of type I IFNs increases, and this may have suppressive effects on inflammation via upregulation of tristetraprolin (73). In addition, narrow-band UVB treatment can be used as a treatment for some inflammatory skin diseases, such as psoriasis, and can result in downregulation of IL-17, IL-12, and IFN-regulated pathways (74, 75).

In patients with autoimmune diseases, however, UV light may trigger inflammatory responses. This may be due in part to chronic overexpression of type I IFNs. Increased levels of type I IFNs found in SLE patients correlate with systemic disease activity and severity (76). Further, circulating IFN activity also correlates with cutaneous disease activity in CLE patients (77). Supporting a role for type I IFN in SLE skin, a recent trial of anifrolumab, which blocks type I IFN receptor signaling, shows promise for improvement in CLE lesions (78). At baseline, SLE patients demonstrate an increased IFN signature in their “healthy” keratinocytes (79), likely mediated by chronic overproduction of IFNκ (34, 80, 81). In the skin, type I IFNs stimulate chemokine production and activate adaptive immune responses (82). Indeed, supernatants from SLE>control keratinocytes treated with UVB stimulate the activation of dendritic cells in an IFN-dependent manner (34). Further IFN gene expression in the epidermis correlates with upregulation of the adhesion molecules E-selectin and ICAM-1 that enhance T cell and macrophage recruitment into the skin (83–86). Type I IFNs may also come from non-epithelial sources, including plasmacytoid dendritic cells (pDCs: see below). Further, genomic DNA from UV-irradiated epithelial cells can induce primary human monocytes to secrete more IFNα than those exposed to DNA from non-irradiated epithelial cells (18). This suggests that a UV-induced modification of DNA is at least partially responsible for upregulation of type I IFNs. Lending more support to this idea, colocalization of 8-OHG and MxA, an IFN-upregulated gene, is seen in the epidermis of UV-induced LE lesions (18).

Integration of the 8-OHG and IFN response may occur via stimulator of interferon genes (STING). STING coordinates signals from cytoplasmic DNA sensors, and is negatively regulated by the pro-autophagic protein unc-51-like kinase 1 (ULK1) (87). Upon UV-induced DNA damage, ULK1 stability is disrupted by the loss of the activating molecule in Beclin-1-regulated autophagy (AMBRA1) (88). The resulting increase in STING activity causes activation of interferon regulatory factor 3 (IRF3), potentiating type I IFN secretion and exacerbating autoimmunity in response to UV exposure (88).

Another contributor to skin interferons may be the lupus band, which consists of nuclear debris, complement, DNA and IgG and IGM autoantibodies and is induced by ultraviolet light (89). Positive lupus band testing is found at the dermo-epidermal junction in many systemic and cutaneous lupus patients (90, 91), and its presence positively correlates with disease activity (92). Because immune complexes stimulate IFNα production by pDCs (93), and this is further positively regulated by inflammatory cells present in lupus skin (94–96), UV-induced immune complexes may contribute to photosensitive responses. In addition, immune complexes stimulate inflammasome activation (97, 98) and expansion of B cell subsets (99), which may amplify the inflammatory response in the skin once started.

Elevated levels of pro-inflammatory cytokines, including IL-6, TNFα, and IL-1β, in SLE patients are associated with increased disease activity (100). UVB irradiation has been shown to further increase levels of TNFα in normal human keratinocytes, likely mediated through upregulation of IL-1α, (101, 102). UVB exposure induces more IL-6 production from SLE keratinocytes compared to those from healthy controls (81). This difference is driven by increased production of type I IFNs, as control keratinocytes treated with type I IFNs increase their IL-6 production, while lupus keratinocytes treated with type I IFN blockade have decreased IL-6 production (81). More specifically, keratinocyte specific secretion of IFNκ increases after UVB treatment of lupus keratinocytes and neutralization of this type I IFN abrogates IL-6 production (81). Additionally, increased IL-1β and TNFα expression promotes release of inflammatory chemokines CCL5, CCL22, CXCL8, and CCL27 by epidermal keratinocytes and this may support leukocyte recruitment, especially memory T cells, into the skin following UV exposure (82).

Tumor necrosis factor- (TNF-) like weak inducer of apoptosis (TWEAK) and its receptor fibroblast growth factor-inducible 14 (Fn14) play a role in modulation of inflammatory responses in the skin by activating NFκB in keratinocytes (103). Activation of the TWEAK-Fn14 signaling pathway is significantly increased in lesional skin of SLE patients. Additionally, mRNA expression of TWEAK, Fn14, and several genes turned on by this pathway including CCL5, monocyte chemoattractant protein-1 (MCP-1) and CXCL10 is higher in these lesions compared to healthy controls (103). Overlap of Fn14 and Ro52 is observed in the upper epidermis of lesional skin suggesting a possible role for TWEAK-Fn14 activation in Ro-52 mediated photosensitivity of CLE patients, similar to what has been observed in mouse models (104).

#### Immune cell recruitment

UV exposure induces recruitment of innate and adaptive immune cells to the skin. Neutrophils are one of the first cell populations recruited to healthy skin after UV exposure. Once present, they secrete IL-10 which provides immunosuppressant effects (105). Intriguingly, in photosensitive disorders, such as polymorphic light eruption, recruitment of neutrophils is diminished and it is hypothesized that the immunosuppressive functions of neutrophils are subsequently lost (106). Localized O_2_ depletion by infiltrating neutrophils undergoing respiratory bursts is important for resolution of mucosal inflammation; therefore, loss of this hypoxic environment resulting from decreased neutrophil recruitment may play a role in the increased inflammation seen in lupus skin (107). In CLE lesions, neutrophils have been shown to release neutrophil extracellular traps “NETs” which may participate in tissue damage (108). These NETs are interferonogenic and may contribute to pro-inflammatory, IFN rich environment in lupus skin lesions (109).

Neutrophils from SLE patients have a lowered ability to produce ROS when compared with healthy controls and this decrease correlates with disease severity (110). Polymorphisms in *Ncf1*, a gene encoding a component of the NADPH oxidase complex, are found in SLE patients and these are associated with decreased ROS generation in neutrophils and an increase in expression of type I IFN regulated genes (111). It is not yet known if UV irradiation further affects the capacity of lupus neutrophils to produce ROS. Additionally, in MRL/*lpr* lupus-prone mice, treatment with MitoTEMPO, a mitochondrial ROS scavenger, results in decreased neutrophil NETosis, immune complex deposition in the kidney, and type I IFN production; however, the effect in UV-irradiated skin is not known (109).

PDCs are a subset of dendritic cell shown to accumulate in cutaneous lupus lesions and locally produce IFNα (112). UV triggers production of CXCL9, CXCL10, and CXCL11 chemokines that attract pDCs and other inflammatory cells (82). Following UV exposure, pDCs accumulate at the dermoepidermal junction to a greater extent in SLE patients vs. healthy controls (80). Increased translocation of autoantigens such as RNA and DNA fragments by UV can result in formation of immune complexes that can subsequently be internalized via FcγRII on pDCs, activate endosomal TLR7/9, and induce IFNα production by the pDC (113–115). This initiates an amplification loop in which IFNα further promotes chemokine and IFNκ expression in the skin, recruiting additional leukocytes, and furthering inflammation that contributes to cutaneous lesion development (34, 82).

Mast cells may also be involved in UV responses in the skin. The number of mast cells in CLE skin lesions is significantly higher than in normal skin and even higher in sun-exposed diseased skin compared to sun-protected diseased skin. Recruitment of mast cells, which have been shown to produce matrix metalloproteinases (MMPs), can be induced by IL-15 and CCL5 (116–119). MMPs are a family of enzymes secreted by a variety of cell types that are known to play a crucial role in processes ranging from tissue degradation and repair to apoptosis and inflammation (120). Sera of lupus patients often have elevated levels of several MMPs and lower levels of tissue inhibitor of metalloproteinases (TIMP)-1 compared to healthy controls (121–124). TIMP-1 is also shown to be downregulated in LE skin lesions while TIMP-3, which may promote keratinocyte apoptosis, is upregulated (125, 126). Together, this suggests that UV light may promote mast cell recruitment and MMP production that may be further exacerbated in lupus skin (127).

T cells are also recruited after UVB exposure. Skin resident T cells have a protective role in limiting DNA damage after UVB exposure (128). UVB-mediated activation of regulatory T cells may participate in immunosuppressive effects of UV light (129). Intriguingly, a recent report suggests that T cells may have innate photosensing abilities that discriminate the wavelength of light and in turn modulate chemotactic responses (130). In lupus patients, UV exposure results in accumulation of T cells at the dermoepidermal junction during lesion onset and this infiltration persists in later lesions (131, 132).

### Circadian clock and UV-induced skin inflammation

The circadian clock is a recently understood mechanism that regulates many physiological processes including those of the immune system. A recent study showed that circadian clock-controlled cryptochromes (CRY) 1 and 2 are differentially expressed in narrow band-UVB irradiated human skin with lower levels of CRY2 associating with increased erythema (133). It may be that CRY2 plays a role in protection against skin damage caused by UV exposure (133). CRY2 is involved with regulation of c-MYC degradation and, therefore, may abrogate UV-induced keratinocyte apoptosis (134). It is intriguing to surmise that pathogenesis and photosensitivity of SLE patients may be partially explained by decreased CRY2 expression that inhibits protection against UV, however, further studies will need to be carried out to determine whether disease is associated with differential cryptochrome expression. Studies in mice have also suggested the circadian clock may be a contributing factor in autoimmunity (135).

### Wild type and autoimmune murine models of UV exposure

Although lupus patients experience sensitivity to UV exposure and display both local and systemic flares, understanding the mechanism is challenging due to variability between patients (136). Thus, murine models are ideal for understanding the mechanisms regulating both the local and systemic UV response with the caveat that no one animal model will mimic every aspect of human disease perfectly. Like in humans, UVA has shown therapeutic effects for autoimmune conditions in mice (137). However, most studies that examine the mechanism behind UV damage utilize UVB; thus, mechanisms involved in local and systemic response following UVB treatment will be reviewed below.

### UV-induced DNA damage and apoptosis

Similar to humans, mice also exhibit increased apoptosis and DNA damage in the skin after UVB exposure. In murine skin, keratinocytes and fibroblasts are susceptible to UVB-induced apoptosis (138–141). Both TLR and TWEAK-Fn14 signaling pathways have been shown to regulate this process. TLR 4-MyD88 signaling pushes cells to undergo apoptotic vs. necrotic cell death pathways after UVB exposure via caspase 3 activation, as mice deficient for either TLR4 or MyD88 display increased necroptosis markers and TNFα production (142). The TWEAK-Fn14 signaling pathway has also been investigated in mice for its role in apoptosis, since Fn14 is upregulated on keratinocytes following UVB exposure. Knockout (KO) of Fn14 led to protection from UVB induced skin inflammation (143), while the addition of TWEAK led to increased apoptosis of keratinocytes from UV treated MRL/*lpr* mice (144). UV exposure also led to increased DNA damage/release in both wild-type mice and lupus-prone mice, though lupus-prone MRL/*lpr* mice demonstrate increased susceptibility to UV-mediated DNA release (145). This UV induced DNA damage may play a role in lesion development, as TREX1 KO mice, which lack cytosolic DNase, develop lupus-like lesions (146). Further, UV-modified DNA can induce CLE-like lesions when injected into the skin of MRL/*lpr* mice (18). These data suggest a role for TLR and TWEAK-Fn14 signaling in mediating increased apoptosis within the skin of lupus-prone mice following UVB exposure. Also the increased DNA damage following UVB exposure plays a part in lesion development. Though DNA damage and apoptosis result from UV irradiation, the differences between wild-type mice and lupus-prone mice regarding mechanisms of immune activation remains understudied.

### UV-induced autoantigen exposure

Exposure of autoantigens at the dermoepidermal junction also occurs in lupus-prone mice following UVB treatment. One study identified immune complexes and antibodies to desmoglein 3 at the dermoepidermal junction in NZB/NZW F_1_ mice exposed to 500 mJ/cm^2^ UVB every other day (147). While production of anti-Ro antibodies is rare in murine lupus models, UVB induces similar externalization of the Ro autoantigen in mice. Indeed, injection of Ro+ serum from patients with subacute cutaneous lupus into Balb/c mice exposed to UVB results in deposition of anti-Ro antibodies at the dermoepidermal junction (148). Further studies should address the role of autoantibodies in murine lupus models of UV-mediated skin inflammation.

### UV-induced inflammation

#### Cytokines

Murine cytokine production after UVB is similar to that seen in human skin: TNFα, IL-6, IL-1, IL-23, and type I IFNs are all increased (139, 142, 149). Most of the cytokine induction is fairly rapid: TNFα and IL-6 production occurs 8–24 h after UVB exposure (150). However, data examining their role in UVB-mediated changes remain limited. In lupus-prone mice, IFN-regulated gene *Ifi202* has a pro-inflammatory effect on apoptosis following UVB stimulation (151), but in wild-type mice, IFNs demonstrate a protective effect in the skin as mice lacking the type I IFN receptor have greater inflammatory responses (152). UVB induces colony-stimulating factor-1 (CSF1) which likely enhances macrophage recruitment to the skin (153). Following UVB, TNFα has a pro-inflammatory effect by increasing apoptosis of keratinocytes (149, 154, 155). Though studies on the role of IL-1 family members following UV exposure are limited, mice transgenic for IL-1α demonstrate skin inflammation (156). IL-6 ^−/−^ mice demonstrate decreased epidermal proliferation after UVB and also decreased systemic IL-10, suggesting IL-6 may have both epidermal and immune regulatory functions (157). IL-23 in wild-type mice has a protective effect on UVB-mediated damage by reducing DNA damage and increasing T regulatory cells (158); however the function of this cytokine has not been examined in lupus-prone mice following UVB stimulation. Intriguingly, neutralizing antibodies to IL-23 have a protective effect in lupus-prone mice, which suggests a pro-inflammatory function for this cytokine after UVB stimulation (159). Further exploration into the role of these cytokines following UVB exposure in wild-type and lupus-prone mice may yield novel data for therapeutic development for photosensitivity.

#### Immune cell recruitment

Epidermal damage from UVB exposure results in upregulation of chemokines and recruitment of neutrophils, monocytes, macrophages, dendritic cells and T cells (140, 143, 160). The dose of exposure regulates the inflammatory response. Hairless mice exposed to low dose (20 mJ/cm^2^) UVB demonstrate increased epidermal thickness but not inflammation. The same mice exposed to a single high dose (400 mJ/cm^2^) demonstrate neutrophil and macrophage recruitment (161). C57BL/6 mice exposed to two doses of 500 mJ/cm^2^ of UVB also demonstrate infiltration of pDCs within 24 h and macrophages and neutrophils after 24–78 h (162). In wild-type mice, CD4^+^ T cells and CD8^+^ T cells exhibit pro-inflammatory functions through production of IFNγ following UVB stimulation (160); this inflammation is downregulated via induction of T regulatory cells in the skin (163). IFNα-producing monocytes are recruited to the skin in wild-type mice following UVB exposure, and they also exhibit a negative regulatory effect on UVB-driven inflammation via type I IFN-mediated pathways (152). Resident Langerhans cells are essential to resolution of UVB induced skin inflammation through their phagocytosis of apoptotic keratinocytes (160) and through promotion of epidermal growth factor receptor signaling (164); thus, they also exhibit an anti-inflammatory role.

The effect of UVB in mice with a propensity for autoimmune conditions is less well-studied. In lupus-prone MRL/*lpr* mice, markers of neutrophil and macrophage infiltration are present after UVB, but how this compares with wild-type mice was not evaluated (143). Other studies have compared effects in lupus-prone vs. wild type mice. Increased CD8^+^ and CD4^+^ cells were noted in MRL/*lpr* vs. Balb/c mice after 2 and 6 days of 500 mJ/cm^2^ UVB treatment (153). Production of chimerin and recruitment of pDCs to the skin after UVB exposure is increased in MRL/*lpr* vs. wild-type mice (162). *Ex vivo* irradiation of lymph nodes from lupus-prone (both NZB/NZW F_1_ and MRL/*lpr*) vs. wild-type mice exhibited greater upregulation of ICAM-1 and LFA-1, which promotes migration of immune cells into the tissues (139). These studies have generated a preliminary understanding of the differential effects of UVB in lupus-prone vs. wild-type mice, but additional research is needed.

### UV-induced systemic disease flares

Anecdotal and case report data support a link between cutaneous UVB exposure and induction of systemic disease flares in patients (5, 6). This connection between the cutaneous and systemic immune system has not been well characterized in human or murine models [reviewed in (165)]. To date the main lupus-prone mouse model that has demonstrated systemic responses to UV is BXSB male mice, which carry an additional copy of TLR7 as part of the Yaa locus (166). In this strain, daily exposure to 400 mJ/cm^2^ full spectrum UV for 1 week resulted in 66% of mice succumbing to death after 2 weeks. This level of irradiation did not impair survival in Balb/c, MRL/*lpr* or (NZBxNZW)F_1_ mice. Chronic exposure to 120 mJ/cm^2^ thrice weekly also resulted in >85% lethality after 4 weeks of treatment in male BXSB mice. Death in the male BXSB mice was accompanied by changes consistent with lupus nephritis (166). Whether it is TLR7 driving this phenotype has not been elucidated, but stimulation of TLR7 in Balb/c mice with topical TLR7 agonist for 2 weeks followed by UVB resulted in rising autoantibody titers compared to UVB only-treated mice (167), and TLR7 stimulation itself can promote systemic disease flares(168). This suggests that TLR7 signaling may have a role in UVB-mediated systemic immune activation. However, epidermal damage itself may be sufficient to drive lupus flares in lupus-prone mice (169), so the effects of UVB on systemic immune activation may be multivariate.

Sensing of UVB-modified nucleic acids may contribute to systemic flare development following UVB exposure. For example, injection of UVB-induced apoptotic DNA in wild-type and lupus-prone MRL/*lpr* mice led to development of lupus-like characteristics such as increased dsDNA antibodies and proteinuria (170, 171). Hypomethylation of DNA seems to be important for this process (171). It is tempting to speculate that these systemic effects may be secondary to STING activation as UVB-modified DNA is resistant to degradation by TREX1 and is able to induce IFN responses and cutaneous lupus-like lesions when injected into the ear of MRL/*lpr* mice (18). Further exploration is needed to understand the role of UVB-mediated DNA changes in driving systemic immune responses in SLE.

## Summary

**Figure 1:**
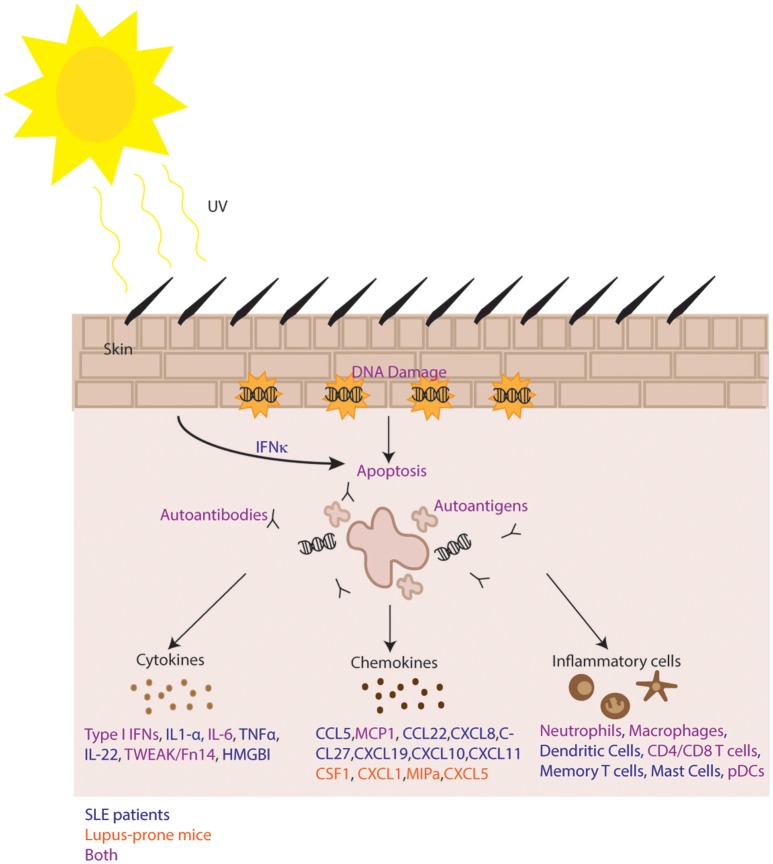
Summary of mechanisms of photosensitivity. In lupus, increased IFN kappa promotes UV-mediated apoptosis resulting in immune complex formation, autoantigen exposure and release of numerous inflammatory cytokines and chemokines. Infiltration of inflammatory cells follows and is perpetuated by inhibition of negative regulatory mechanisms. Pathways with evidence in both human and murine systems are shown in purple. Human only pathways are shown in blue, and murine-specific pathways are shown in orange.

UV irradiation leads to a complex sequence of events in the skin that generates varied inflammatory changes depending on the target (summarized in Figure 1). UV exposure triggers ROS production, DNA damage, and apoptosis that can result in autoantigen translocation to the surface of keratinocytes where they are exposed for immune recognition by autoantibodies. Impaired or inflammatory clearance of these apoptotic cells in SLE patients may occur due to decreased levels of complement proteins and altered complement function. UV exposure modifies DNA and also activates STING to increase production of type I IFNs and other pro-inflammatory cytokines and chemokines that promote leukocyte recruitment into the skin, further enhancing disease progression and lesion formation. While there is a growing body of knowledge regarding type I IFNs and lupus, the specific sources of these IFNs in the skin as well as the roles they play in processes such as UV-induced apoptosis and immune system activation are yet to be fully understood. Additionally, due to limited knowledge of the changes induced in immune cell populations following UV exposure of lupus patients and lupus-prone mice, further studies will need to elucidate the specific mechanisms that may be at play.

## Author contributions

SJW, SNE, JEG, and JMK participated in the writing and editing of the manuscript.

### Conflict of interest statement

JMK is an Associate Editor for *Frontiers in Immunology*. The remaining authors declare that the research was conducted in the absence of any commercial or financial relationships that could be construed as a potential conflict of interest.

## Abbreviations

CLE: cutaneous lupus erythematosus
IFN: interferon
pDCs: plasmacytoid dendritic cells
ROS: reactive oxygen species
SLE: systemic lupus erythematosus
TLR: toll-like receptor
UV: ultraviolet.
